# Isotropic Nature of the Metallic Kagome Ferromagnet Fe_3_Sn_2_ at High Temperatures

**DOI:** 10.3390/cryst11030307

**Published:** 2021

**Authors:** Rebecca L. Dally, Daniel Phelan, Nicholas Bishop, Nirmal J. Ghimire, Jeffrey W. Lynn

**Affiliations:** 1 NIST Center for Neutron Research, National Institute of Standards and Technology, Gaithersburg, MD 20899-6102; 2 Materials Science Division, Argonne National Laboratory, Argonne, Illinois 60439; 3 Department of Physics and Astronomy, George Mason University, Fairfax, VA 22030; 4 Quantum Science and Engineering Center, George Mason University, Fairfax, VA 22030

**Keywords:** inelastic neutron scattering, topological materials, anomalous Hall effect, isotropic ferromagnet, kagome, frustrated magnetism, skyrmion

## Abstract

Anisotropy and competing exchange interactions have emerged as two central ingredients needed for centrosymmetric materials to exhibit topological spin textures. Fe_3_Sn_2_ is thought to have these ingredients as well, as it has recently been discovered to host room temperature skyrmionic bubbles with an accompanying topological Hall effect. We present small-angle inelastic neutron scattering measurements that unambiguously show that Fe_3_Sn_2_ is an isotropic ferromagnet below $T_{C}\approx 660\;\;\text{K}$ to at least 480 K – the lower temperature threshold of our experimental configuration. Fe_3_Sn_2_ is known to have competing magnetic exchange interactions, correlated electron behavior, weak magnetocrystalline anisotropy, and lattice anisotropy; all of these features are thought to play a role in stabilizing skyrmions in centrosymmetric systems. Our results reveal that at elevated temperatures, there is an absence of magnetocrystalline anisotropy and that the system behaves as a typical exchange ferromagnet with a spin stiffness $D\left(T=0\;\text{K}\right)=271\pm 9\;\text{meV}\;\;{\text{Å}}^{2}$.

## 1. Introduction

**Figure 1.**
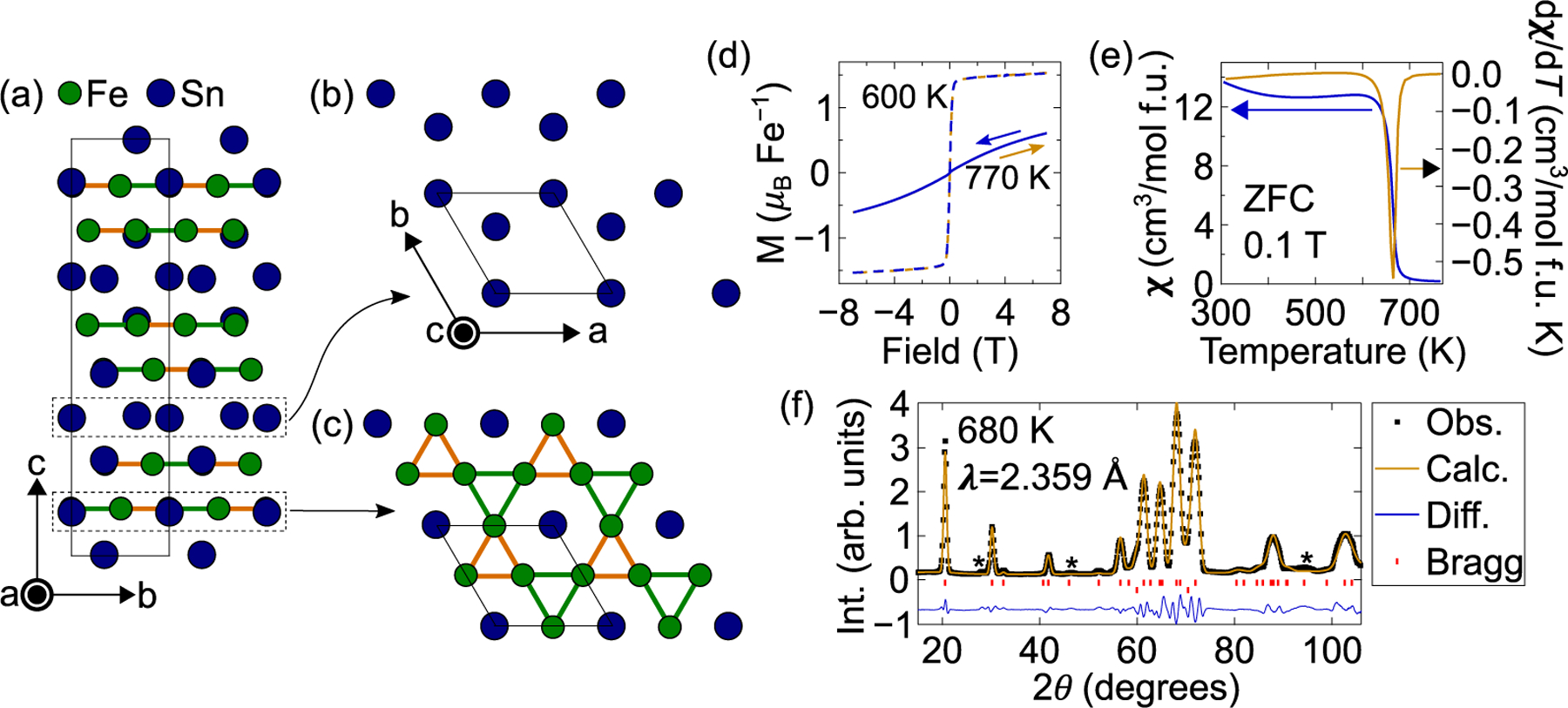
Crystal structure and characterization of Fe_3_Sn_2_. The crystallographic space group is $R\bar{3}m$ with reported lattice parameters a=b=5.344 Å and c=19.845 Å. [8] (**a**) View along the **a**-axis. The solid black line represents the unit cell and Fe atoms are shown as the smaller green circles and Sn atoms are shown as the larger blue circles. A single Fe site is offset from any high symmetry position (x/a=0.4949, y/b=0.5051, and z/c=0.1134) leading to two different Fe-Fe bond lengths in the ab-plane (the so-called “breathing” kagome). Two Fe-Fe bond lengths are shown, where the shorter bond is in orange, and the longer bond is in green. (**b**) A Sn-only layer viewed along the **c**-axis, where the Sn atoms are arranged on a honeycomb lattice. (**c**) An Fe-Sn layer viewed along the **c**-axis, showing the breathing kagome lattice made up of Fe atoms. The axes labels for (**c**) are the same as in (**b**), and the parallelogram outlined by a solid black line for both panels represents the unit cell. (**d**) Magnetization measurements taken at 770 K (solid lines) and 600 K (dashed lines). The samples show no signs of coercivity as the sweep down in field (blue lines) coincides with the sweep up (orange lines) in field. (**e**) Zero-field cooled (ZFC) magnetic susceptibility measurement taken in a 0.1 T applied magnetic field. The derivative (right axis) clearly shows the ferromagnetic transition at $T_{C}\approx 665\;\text{K}$. (**f**) Neutron powder diffraction data taken above the magnetic transition at 680 K. The data demonstrate the structure is consistent with that reported. The upper set of red tic marks denote Fe_3_Sn_2_ Bragg peak positions and the lower set denote Al Bragg peak positions coming from the sample canister. A few small impurity peaks were observed but not identified, and these are marked by the * symbol.

The two-dimensional kagome lattice lends itself to hosting a variety of phenomena depending on the chemical species occupying the network of corner-sharing triangles. For example, the tight-binding model for itinerant electrons leads to a an electronic spectrum with a flat band and two Dirac crossings at the symmetry protected *K* and *K′* corner points of the hexagonal Brillouin zone. Chemical tuning can drive the Fermi level to meet the Dirac points (a Dirac semimetal) to realize chiral massless charge carriers like that in graphene. [1,2] The prediction of the flat band – on the extreme opposite from a Dirac band – is the result of destructive interference of Bloch waves from the lattice geometry. Consequently, this nontrivial flat band can exhibit interesting physics such as flat-band ferromagnetism and a finite Chern number. Experimentally, FeSn was shown to host both flat bands and Dirac fermions [3] due to the isolated Fe kagome layers rendering it a nearly perfect realization of 2D kagome physics. Fe_3_Sn_2_ is similar in structure, but features isolated breathing kagome bilayers, as shown in Fig. 1. Interestingly, the bilayers and breathing structure were still theorized to have a band structure with similar features. Instead of one Dirac crossing at each *K* and *K′* point, there are two which are symmetric about each point, [4] and the fermions are both spin-polarized due to the breaking of time-reversal symmetry and massive due to the opening of a gap from spin-orbit coupling. The combination of these effects gives rise to a non-zero Berry curvature which is consistent with the quadratic relationship of the anomalous Hall resistivity (AHE) with the longitudinal resistivity, [5] implying the intrinsic Karplus and Luttinger mechanism [6] is responsible for the large AHE. It was also recently shown that nearly flat bands near the Fermi surface exist, which may contribute to the observed high-temperature ferromagnetism. [7]

Metallic kagome ferromagnets clearly exhibit elegant physics, however, they are elusive with only two being reported: Co_3_Sn_2_S_2_, a semimetal, [9] and the aforementioned Fe_3_Sn_2_. As alluded to thus far, the electronic structure has signatures of non-trivial topology, but recently, Fe_3_Sn_2_ has garnered growing attention for the discovery of topologically non-trivial spin textures. The observation of room temperature skyrmion bubbles [10] quickly led to reports of nanostructured skyrmionic devices [11–14] and studies of the associated properties such as the topological Hall effect [15–17] and skyrmion thermopower. [18] The space group of Fe_3_Sn_2_ is the centrosymmetric $R\bar{3}m$, meaning the mechanism for skyrmion bubble formation is not due to the conventional breaking of crystalline inversion symmetry with Dzyaloshinshkii-Moriya interactions found in conventional B20 skyrmion systems. Instead, topological magnetic structures in centrosymmetric systems are due to the presence of anisotropy and/or frustration. The underlying source of each which is needed to stabilize skyrmions has become widely studied in recent years. One common model is the triangular lattice with frustrated Heisenberg antiferromagnetic exchange interactions. [19] Although the magnetic frustration alone was shown to lead to a skyrmion phase, either lattice and/or spin anisotropy [20], particularly easy-axis anisotropy, [21,22] was shown to be helpful in stabilizing the skyrmions.

From a frustrated magnetism perspective, Fe_3_Sn_2_ has been of interest for quite some time. Original neutron powder diffraction measurements indicated collinear ferromagnetic order below the onset of magnetism at $T_{C}\approx 660\;\;\text{K}$ with moments oriented along the *c*-axis. [23] A spin-reorientation transition to the ab-plane starting below 250 K was identified and later measurements implied a slightly non-collinear structure was more likely starting below 300 K [24] and that the spin-reorientation transition was actually first-order in nature and occurs at ≈150  K. [25,26] The non-collinearity is thought to be due to frustrated magnetic exchange, and would also explain the large anomalous Hall effect [5,27] and possibly some of the temperature regimes where the topological Hall effect is observed if the scaler spin chirality is finite. Bulk magnetic measurements have also shown Fe_3_Sn_2_ to be an extremely soft ferromagnet at all temperatures with no coercivity, implying any easy-axis magnetic anisotropy must be very weak.

Here, we present our small-angle inelastic neutron scattering study of the magnetic excitations in Fe_3_Sn_2_ between 480 K and 660 K and unambiguously show that no significant spin wave gap is observed within experimental uncertainties between these temperatures. Below 480 K, the spin stiffness parameter, $D\left(T\right)$, becomes too large and the spin wave full-width-at-half-maximum in energy, $\text{Γ}\left(q\right)$, has narrowed to the point that the excitations move outside our measurement window. However, our results show that down to at least 480 K, Fe_3_Sn_2_ behaves as an ideal isotropic ferromagnet, and any onset of significant magnetic anisotropy that may contribute to the topological spin textures must develop below this point.

## 2. Materials and Methods

Polycrystalline samples of Fe_3_Sn_2_ were synthesized by solid state reaction. Stoichiometric amounts of Fe powder (Alfa Aesar 99+%) and Sn powder (Alfa Aesar 99.995%) were mixed and pelletized. The pellet was sealed in a fused silica ampoule under vacuum. The sealed ampoule was heated to 800° C at the rate of 1° C/hour and was kept at 800° C for 1 week. After 1 week, the ampule at 800° C was quenched into ice water. The pellet was reground, re-pelletized, and sealed into the fused silica ampoule under vacuum and was annealed at 800° C for 1 week.

Magnetic measurements were performed on a piece of pressed pellet of Fe_3_Sn_2_ powder using a Quantum Design MPMS DC-SQUID magnetometer using the oven insert between 300 K and 756 K.

Neutron powder diffraction (NPD) measurements were taken using the triple-axis spectrometer, BT-7, at the NIST Center for Neutron Research. [28] A 17 g sample of polycrystalline Fe_3_Sn_2_ was sealed in a cylindrical aluminum canister, which was mounted inside a closed cycle refrigerator. Data were collected in two-axis mode using a position sensitive detector. Söller collimators of 50*′ −* 40*′ R* were used before and after the sample, respectively (where *R* indicates radial). Data were refined using the Rietveld method and the program, FullProf. [29]

Inelastic neutron scattering data were also taken using BT-7 and the same 17 g sample as in NPD. Two different small-angle inelastic neutron scattering configurations were used in order to obtain data over a wide temperature range. For higher temperatures (630 K to 660 K), PG(002) monochromator crystals with vertical focusing and PG(002) analzyer crystals were used, and constant-Q scans were taken with a fixed incident energy of 13.7 meV. Söller collimators of 10*′* – 10*′* – 10*′* – 25*′* were used before the monochromator, sample, analyzer, and detector, respectively, and the vertical resolution was measured using a graphite crystal and found to be $0.16\;{\text{Å}}^{-1}$. For lower temperatures (480 K to 610 K), PG(004) monochromator crystals with vertical focusing and PG(004) analzyer crystals were used, and constant-Q scans were taken with a fixed incident energy of 35 meV. The same collimations as the $E_{i}=13.7\;\text{meV}$ experiment were used and the vertical resolution was found to be $0.24\;{\text{Å}}^{-1}$. The same scans taken at high temperatures were also taken at much lower temperatures (300 K for the $E_{i}=13.7\;\text{meV}$ experiment and 250 K for the $E_{i}=35\;\text{meV}$ experiment), and these data were used for background subtraction.

**Figure 2.**
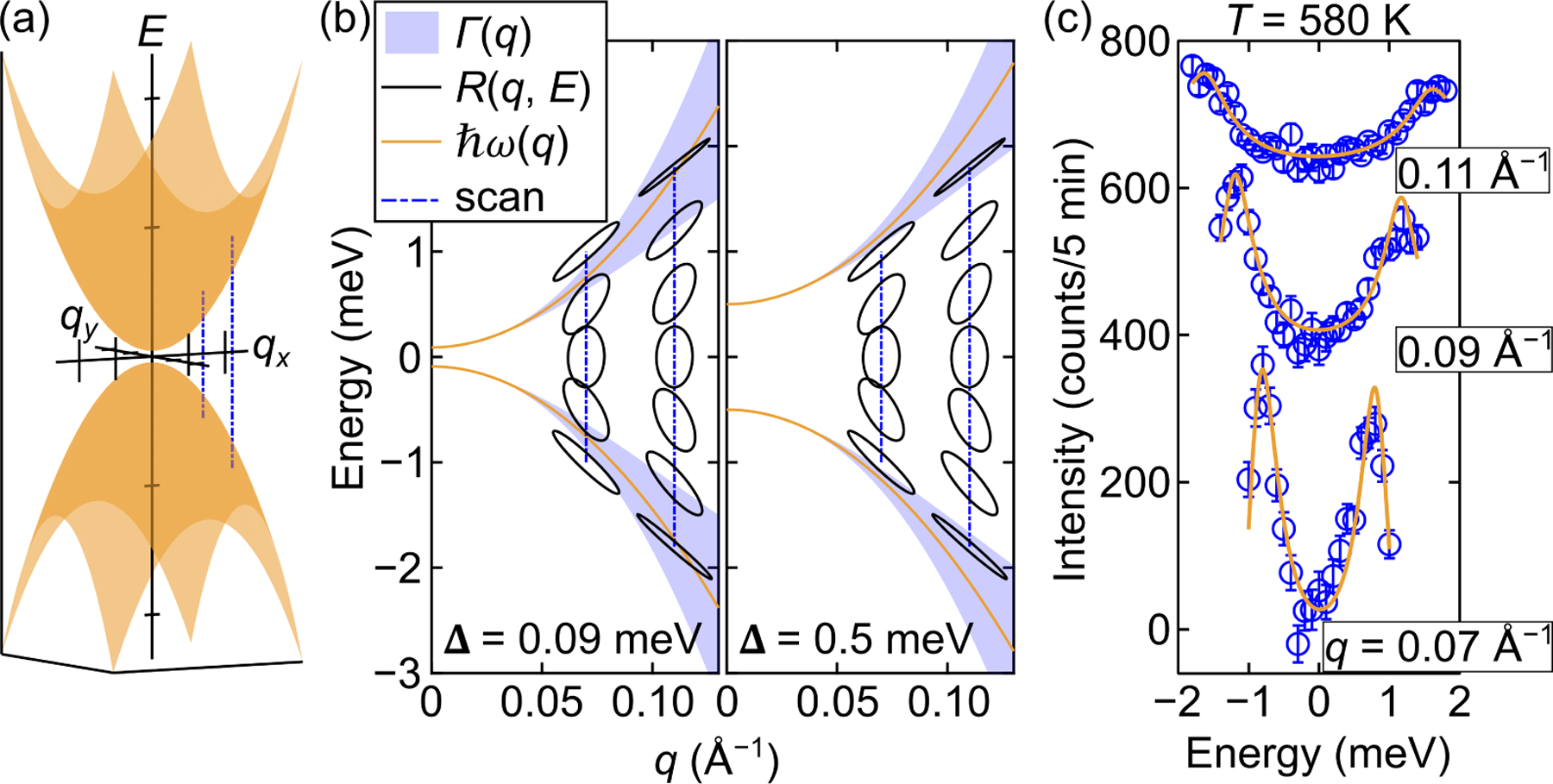
(**a**) A three-dimensional schematic of an isotropic parabolic spin wave dispersion near Q=0 and energy transfer, E=0. The dispersion is shown as the orange surface, and dashed blue lines show the direction of constant-Q scans cutting through the excitation along E. (**b**) A two-dimensional schematic demonstrating how the experiment captures the intensity from the spin wave excitations. The orange solid line represents the dispersion, $ћ\omega \left(q\right)=\text{Δ}+D\left(T\right)q^{2}$, and the surrounding blue surface represents the full-width-at-half-maximum of the spread in energy of the dispersion, $\text{Γ}\left(E\right)$, due to thermally induced magnon-magnon interactions. Dashed blue lines are examples of constant-Q scans made in the experiment. Overlayed on these lines are the instrumental resolution ellipses, $R\left(Q,E\right)$, along $Q=0.07\;{\text{Å}}^{-1}$ and $0.11\;{\text{Å}}^{-1}$. The left panel used the refined values for the dispersion from the actual data at T=580K in the $E_{i}=35\;\text{meV}$ experiment. The right panel used the same parameters, but increased the gap to be 0.5 meV in order to demonstrate the sensitivity of the technique to the size of the gap. Here, the scans performed during the experiment wouldn’t be able to reach the signal of the spin waves. (**c**) The actual data at T=580K in the $E_{i}=35\;\text{meV}$ experiment. The solid orange lines are the refined fits to the data, shown as blue circles. The three constant-Q scans are vertically offset from one another for clarity.

In the small-angle inelastic neutron scattering configuration, spin waves are probed in the long-wavelength (i.e. small-*q*) limit, and the dispersion for a ferromagnet is

(1) $$ћ\omega \left(q\right)=\text{Δ}+D\left(T\right)q^{2},$$

where Δ is any anisotropy gap and $D\left(T\right)$ is the spin wave stiffness which in mean field theory is proportional to the magnetization. The kinematic constraints for the scattering severely restrict the range of energy transfers accessible, so that the spin waves can only be observed if there is little to no anisotropy gap. The point in reciprocal space where the spin waves are being probed can be viewed by the schematic in Fig. 2(a). Here, a parabolic dispersion about Q=0 and energy transfer, E=0, is shown. About this point, the dispersion of an isotropic ferromagnet powder sample is identical to that of a single crystal. Similar experiments on amorphous alloys [30] and powder samples of manganites [31] have been widely used to establish their isotropic nature.

One advantage of studying the spin waves in the long-wavelength limit (i.e. about Q=0 ) is that the instrumental resolution is focused on both the energy gain and energy loss side, unlike about a Bragg point where there is a focused and de-focused side. More details on the resolution function in the small-angle limit can be found in Ref. [32] The intensity detected in a neutron scattering experiment represents a convolution of the instrumental resolution, $R\left(Q,E\right)$, and the scattering function, $S\left(q,ћ\omega \right)$, making it necessary to include the convolution when analyzing the data. Using the Cooper-Nathans approximation for the resolution, we used the program ResLib [33] to fit each set of data (where a set of data consists of all the constant-Q scans at a single temperature) to the scattering function,

(2) $$S\left(q,ћ\omega \right)\propto F\left(q,ћ\omega \right)\frac{ћ\omega }{1-e^{-ћ\omega /k_{B}T}},$$

where $F\left(q,ћ\omega \right)$ is the spectral weight function, $k_{B}$ is the Boltzmann constant, and T is the temperature. At finite temperatures, magnon-magnon interactions lead to damping effects in energy for the spin waves. For Heisenberg ferromagnets below $T_{C}$, and in the energy regime $ћ\omega \ll k_{B}T$, the excitation width in energy has been calculated [34,35] as

(3) $$\text{Γ}\left(q\right)\propto q^{4}T^{2}\left\{\frac{1}{6}\ln^{2}\left(\frac{k_{B}T}{ћ\omega }\right)+\frac{5}{9}\ln\left(\frac{k_{B}T}{ћ\omega }\right)-0.05\right\}.$$

The shape of the broadening in energy is approximated using a Lorentzian function as the spectral weight function, $F\left(q,ћ\omega \right)$, centered about $ћ\omega \left(q\right)$, with $\text{Γ}\left(q\right)$ as the full-width-at-half maximum.

## 3. Results

### 3.1. Characterization

Fig. 1(d) shows the magnetization both above (770 K) and below (600 K) the ferromagnetic transition temperature. No coercivity was observed for either temperature, meaning Fe_3_Sn_2_ is a soft ferromagnet. Fig. 1(e) shows the magnetic susceptibility as a function of temperature at an applied magnetic field of 0.1 T. The derivative of the susceptibility with respect to temperature shows the ferromagnetic transition to be ≈665  K, which is consistent with previous reports that show the Curie temperature to vary anywhere between 640 K and 660 K. [5,16,24]

The observed NPD profile and Rietveld refined fit are shown in Fig. 1(f). The data were taken at 680 K and confirm the structure to be Fe_3_Sn_2_ with refined lattice parameters of a=b=5.3787±0.0004 Å  and c=19.863±0.002 Å. The refined atomic positions for Fe are x/a=0.4940±0.0004, y/b=0.5060±0.0004, and z/c=0.1132±0002. The Sn positions are z/c=0.1039±0.0007 and z/c=0.3318±0.0007 for the Sn1 and Sn2 sites, respectively. The isotropic thermal parameters (*B*) were refined to $0.8\pm 0.1\;{\text{Å}}^{2}$, $4.1\pm 0.5\;{\text{Å}}^{2}$, and $2.6\pm 0.4\;{\text{Å}}^{2}$ for the Fe, Sn1, and Sn2 sites, respectively. There were three small impurity peaks in the pattern that were unable to be identified. They are marked with an * in Fig. 1(f).

### 3.2. Inelastic neutron scattering

We first demonstrate the sensitivity of the small-angle inelastic scattering configuration to the size of the gap in order to discern between isotropic and anisotropic ferromagnets. A schematic of a dispersion following Eqn. 1 near Q= 0 is shown in Fig. 2(a). The neutron scattering plane is defined by two arbitrary orthogonal vectors, $q_{x}$ and $q_{y}$, and constant-Q cuts are shown as blue dashed lines to show how the experimental scans can cut through the dispersion along energy, E.

Each temperature set of constant-Q scans was fit globally to obtain the spin wave parameters, and the parameters for T=580 K were used to create Fig. 2(b). The spin stiffness parameter was found to be $D\left(T\right)=135\pm 3\;\text{meV}\;{\text{Å}}^{2}$ and the gap, Δ=0.09±0.02 meV, where the uncertainties throughout represent one standard deviation due to statistical counting. These parameters were used to create the solid orange line representing $ћ\omega \left(q\right)$. We note that even for an ideal isotropic spin system a small dipolar gap is expected due to ferromagnetic magnetization. The full-width-at-half-maximum of the spin waves in energy, $\text{Γ}\left(q\right)$, is shown as the shaded blue region following Eqn. 3, and the instrumental resolution, $R\left(q,E\right)$, is shown as black ellipses. The maxima and minima of the ellipses along energy represent the allowed scan region which satisfies the required conservation of momentum and energy represented by the scattering triangle (i.e. scanning farther in energy is not possible). Two representative constant-Q scans are shown as dashed blue lines at $\text{0}.07\;{\text{Å}}^{-1}$ and $\text{0}.11\;{\text{Å}}^{-1}$, and with the small gap of 0.09 meV, the center of the instrumental resolution ellipses for both scans is able to pass over the peak of the dispersion on the energy gain and loss sides. This is not possible if the gap is increased to 0.5 meV, as shown in the right panel of Fig. 2(b) (all other parameters from the T=580 K fit were fixed). The actual data from T=580 K are shown in Fig. 2(c) as blue circles and the fits are shown as solid orange lines.

**Figure 3.**
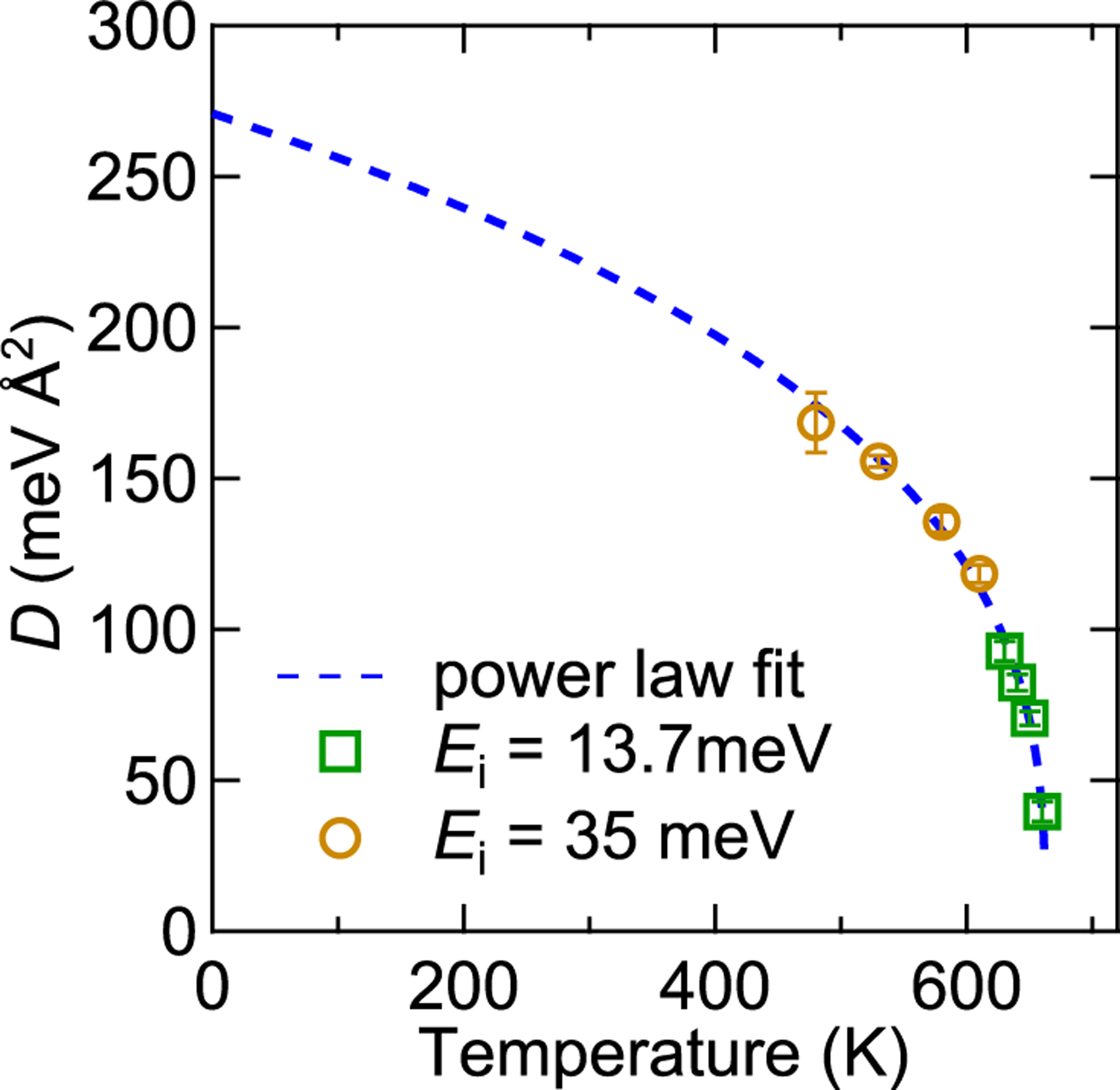
The temperature dependence of the spin wave stiffness parameter, $D\left(T\right)$. Data from both the $E_{i}=13.7\;\text{meV}$ and $E_{i}=35\;\text{meV}$ experiments were included in the power law fit, $D\left(T\right)=D_{0}{\left(\frac{T_{C}-T}{T_{C}}\right)}^{v-\beta }$.

The spin stiffness parameter, $D\left(T\right)$, was extracted from the fits for each temperature and is shown in Fig. 3. The dashed line is a power law fit to the data: $D\left(T\right)=D_{0}{\left(\frac{T_{C}-T}{T_{C}}\right)}^{v-\beta }$, where $D_{0}=271\pm 9\;\text{meV}\;{\text{Å}}^{2}$, $T_{C}=662.4\pm 0.8\;\text{K}$, and v−β=0.34±0.02. The gap was not found to have any meaningful temperature dependence, ranging between 0.06 meV and 0.09 meV, and was the same within plus or minus one standard deviation. It should also be noted that the instrumental resolution in energy for the scans taken is on the order of these values (see Fig. 2(b)), meaning the exact fitted value for the gap is not well-defined. For example, in the $E_{i}=13.7\;\text{meV}$ experiment, the resolution in energy at $Q=0.07\;{\text{Å}}^{-1}$ and E=0 meV is 0.29 meV, and at E=0.6 meV the resolution is 0.07 meV.

## 4. Discussion

The temperature renormalization of the spin stiffness for Heisenberg ferromagnets is expected to follow a power low on approach to $T_{C}$ with the critical exponents v−β=0.34, [36] which is the exponent found in this study, further showing that magnetically, Fe_3_Sn_2_ is a typical exchange ferromagnet at elevated temperatures. In fact, $D_{0}$ and the v−β exponent are strikingly similar to those for elemental Fe [37] and Ni [38], in addition to the amorphous iron magnets already mentioned in Ref. [30], using the same technique.

All ferromagnets have a gap due to the magnetic dipole-dipole interaction between different atoms. [39] This gap is typically small and often out of the range of resolution for inelastic neutron scattering experiments. The dipole-dipole interaction or resolution/instrumental alignment effects are both probable reasons for the observation of a small gap in this study (on the order of 0.06 meV to 0.09 meV ), which is quite small compared to the exchange energy rendering Fe_3_Sn_2_ an isotropic ferromagnet to an excellent approximation. However, the magnetic anisotropy energy due to magnetocrystalline anisotropy was recently calculated to be close to our gap value, at 0.037 meV per Fe atom for the ground state when the easy-axis and spins are oriented within the kagome plane. [40] The ground state for which this value was calculated, though, is in a different temperature regime and spin configuration than that of the present experiment, so it is unclear whether the gap observed is solely due to magnetic anisotropy energy coming from dipolar interactions and/or magnetocrystalline effects.

We now discuss the meaning surrounding the term “anisotropy” in our discussion. Previous studies have cited the uniaxial anisotropy in Fe_3_Sn_2_ as one of the necessary ingredients for the formation of the topologically protected skyrmionic bubbles, [10] and many of the centrosymmetric skyrmion systems discovered thus far are well-known to be a result of competition between frustrated magnetic exchange and spin anisotropy. [21, 22] Unsurprisingly, measurements of the anisotropy energy density, $K_{u}$, have therefore been published [11,14,15] and show that the onset of a magnetic anisotropy precedes the temperatures at which the skyrmion bubbles are found. however, anisotropy can range from preferred orientation of a spin – which all ordered magnets have – to an appreciable energy required to pull spins away from a preferred direction. As a soft ferromagnet, Fe_3_Sn_2_ falls into the former category and can be considered an isotropic ferromagnet in accordance with our results. This is in contrast to the large anisotropies required for permanent magnet devices for magnetostatic energy storage. [41] In fact, one of the appealing properties of skyrmion based devices may be that the weak anisotropy requirements open up the field for potential skyrmion candidate materials, especially when considering inducing small anisotropies into materials via doping is quite common.

An example of a system that internally tunes its anisotropy is Nd_2_Fe_14_B, a hard uniaxial ferromagnet used in permanent magnet applications but also exhibits a spin-reorientation transition like that in Fe_3_Sn_2_. It was found that the rotating spins act to tune the overall anisotropy in the system, [42] although in contrast with Fe_3_Sn_2_, the anisotropy is due to the lanthanide crystal field effect. It’s also instructive to recall that spin anisotropy is not required for skyrmion formation in inversion symmetric systems, [19,20] although these theories have not been specifically applied yet to Fe_3_Sn_2_. Another metallic breathing kagome lattice to host a skyrmion spin texture is Gd_3_Ru_4_Al_12_. [43] In contrast to Fe_3_Sn_2_, the ordered magnetic state is antiferromagnetic and a weak anisotropy is of the easy-plane type. Future work on either of these kagome materials to directly probe the anisotropy gap in proximity to the skyrmion phases would be of interest to explore the role of the anisotropy versus magnetic exchange frustration. Inelastic neutron scattering can be used to achieve this below the temperatures accessible in the work presented here but would require a large mass of single crystals and sub-meV instrumental resolution in a wide-angle scattering experiment.

## Data Availability

Data is available upon request to the corresponding author.
